# HPyV6 and HPyV7 in urine from immunocompromised patients

**DOI:** 10.1186/s12985-021-01496-1

**Published:** 2021-01-22

**Authors:** Carla Prezioso, Marijke Van Ghelue, Ugo Moens, Valeria Pietropaolo

**Affiliations:** 1 Department of Public Health and Infectious Diseases, “Sapienza” University of Rome, Rome, Italy; 2 Microbiology of Chronic Neuro-Degenerative Pathologies, IRCSS San Raffaele Pisana, Rome, Italy; 3 Department of Medical Genetics, Division of Child and Adolescent Health, University Hospital of North Norway, Tromsø, Norway; 4 Department of Clinical Medicine Faculty of Health Sciences, University of Tromsø - The Arctic University of Norway, Tromsø, Norway; 5 Department of Medical Biology, Faculty of Health Sciences, University of Tromsø - The Arctic University of Norway, Tromsø, Norway

**Keywords:** Blood donor, HIV, Human polyomaviruses, Immunocompromised, Multiple sclerosis, PCR, Pregnant women, Psoriasis, Viruria

## Abstract

**Background:**

Human polyomavirus 6 (HPyV6) and HPyV7 are two of the novel polyomaviruses that were originally detected in non-diseased skin. Serological studies have shown that these viruses are ubiquitous in the healthy adult population with seroprevalence up to 88% for HPyV6 and 72% for HPyV7. Both viruses are associated with pruritic skin eruption in immunocompromised patients, but a role with other diseases in immunoincompetent patients or malignancies has not been established.

**Methods:**

PCR was used to determine the presence of HPyV6 and HPyV7 DNA in urine samples from systemic lupus erythematosus (n = 73), multiple sclerosis (n = 50), psoriasis vulgaris (n = 15), arthritic psoriasis (n = 15) and HIV-positive patients (n = 66). In addition, urine from pregnant women (n = 47) and healthy blood donors (n = 20) was investigated.

**Results:**

HPyV6 DNA was detected in 21 (28.8%) of the urine specimens from SLE patients, in 6 (9.1%) of the urine samples from the HIV-positive cohort, and in 19 (40.4%) samples from pregnant women. HPyV7 DNA was only found in 6 (8.2%) of the urine specimens from SLE patients and in 4 (8.5%) samples from pregnant women. No HPyV6 and HPyV7 viruria was detected in the urine samples from the other patients.

**Conclusions:**

HPyV6, and to a lesser extend HPyV7, viruria seems to be common in SLE and HIV-positive patients, and pregnant women. Whether these viruses are of clinical relevance in these patients is not known.

## Background

A virus was isolated in the 1950ies from mouse and because of its ability to induce multiple tumors in animal models named polyomavirus (PyV) [1]. Since then, PyVs have been described in other mammals, including humans, birds, and fish [2, 3]. Members of the *Polyomaviridae* family are characterized by non-enveloped virus particles containing a circular double-stranded DNA genome of approximately 5000 base-pairs (bp). A typical PyV genome codes for the regulatory proteins large T-antigen (LT) and small T-antigen (sT) and at least two capsid proteins, VP1 and VP2. The T-antigens are expressed early during the infection cycle, and are required for viral transcription and replication [4]. These proteins possess oncogenic properties [5–7]. The capsid proteins are expressed after viral replication has initiated and therefore later during infection of the host cell. A non-coding region (NCCR), which encompasses the origin of replication and the transcription regulatory sequences for both the T-antigens and the VPs, separates the coding regions for the T-antigens and capsid proteins [4].

So far, 15 polyomavirus genomes have been isolated from humans. In 1971, BKPyV and JCPyV were the first human PyVs to be described in literature. They were isolated from urine of a renal transplant patient and the brain from a patient with progressive multifocal encephalopathy (PML), respectively [8, 9]. Since then, novel PyVs were originally described in nasopharyngeal aspirates (KIPyV; [10]), bronchoalveolar lavage (WUPyV; [11]), Merkel cell carcinoma (MCPyV; [12]), skin (HPyV6, HPyV7, TSPyV, LIPyV; [13–15]), serum (HPyV9; [16]), genital warts (HPyV10; [17]), feces (STLPyV, QPyV; [18, 19]), liver (HPyV12; [20]), and muscle (NJPyV; [21]). Although HPyVs seem to establish a harmless persistent infection in healthy individuals [22, 23], they may cause diseases in immunocompromised patients. BKPyV is associated with nephropathy in renal transplant recipients and hemorrhagic cystitis in hematopoietic stem cell transplantation [24, 25]. JCPyV causes PML and few cases of JCPyV-associated nephropathy in renal transplant patients have been observed [26–28]. MCPyV is the cause of Merkel cell carcinoma, a neuroendocrine skin cancer [12], and TSPyV is the etiological factor of trichodysplasia spinulosa in immunodeficient patients [29].

Serological studies have demonstrated that HPyV6 and HPyV7 infections are ubiquitous in the healthy adult human population, with a seroprevalence ranging between 74 and 93% for HPyV6 and between 56 and 80% for HPyV7 [30–34]. The presence of HPyV6 and HPyV7 DNA has been examined in different biological samples of healthy controls and different patient groups in a quest to determine the cell tropism and the possible association of these viruses with diseases. HPyV6 and HPyV7 are natural inhabitants of the healthy skin virome [13, 35–39]. These viruses have been detected in other specimens. HPyV6 was present in the urine from one out of 70 patients with hemorrhagic cystitis [40], and HPyV7 was found in urine from one out of 100 liver transplant patients [41] and from five out of 43 hematopoietic stem cell transplant patients [40]. HPyV6, but not HPyV7, was detected in cerebrospinal fluid (CSF) from one out of 243 neurological patients [42] and from one HIV-positive patient with JCPyV-negative progressive multifocal leukoencephalopathy [43]. HPyV6 DNA could be amplified from two out 110 serum samples from HIV-negative patients [44], and from nasopharyngeal aspirates [45] and lymph nodes [46]. HPyV7 DNA was present in the blood from a patient with dyskeratotic dermatosis [47]. Both viruses are also present in eyebrow hair from healthy men [38], and in cervical specimens of women [48].

Clear associations with diseases have not been established, except in severely immunocompromised patients, where HPyV6 and HPyV7 can cause pruritic dermatoses characterized by hyperproliferation of dyskeratotic (with premature or altered differentiation) keratinocytes that result in brownish skin plaques [49–53]. The presence of HPyV6 has sporadically been detected in the skin from lichen simplex chronicus, eosinophilic pustular folliculitis and suppurative foliculitis patients [39, 54]. HPyV6 has also been associated with Kimura disease and patients with dermatopathic lymphadenitis [46, 55]. HPyV6, but not HPyV7 DNA was present in anal/rectal swabs of some men who had sex with men that were HIV-negative, but neither HPyV6 nor HPyV7 were detected in throat/oral swabs and in urethral specimens from these subjects [56].

A role for HPyV6 and HPyV7 in human cancers is lacking although low viral genome copy numbers have been found in few samples of different tumor tissue types [39, 44, 57–69].

To further exploit a possible causative role of HPyV6 and HPyV7 in immunocompromised patients, we examined the presence of DNA from these viruses in urine from systemic lupus erythematosus (SLE), HIV positive, psoriasis, and multiple sclerosis patients, as well as in urine from pregnant women and healthy blood donors. HPyV6 viruria was found in SLE patients, HIV-positive patients and pregnant women, whereas HPyV7 DNA was only detected in SLE and HIV-positive patients. The prevalence of HPyV6 was higher than HPyV7 in these two patient groups. Longitudinal study of the SLE patients showed intermittent viruria.

## Methods

### Patients’ samples

Archival urine samples from five anonymous SLE patients (four women and one man) from Stavanger (Norway) were used. These samples were acquired over a 1-year period. In total, 73 samples were collected [70]. Forty-seven single archival urine specimens from healthy women who were 18–39 weeks pregnant, collected at the university hospital of Northern Norway, have been previously described [70]. The urine samples were stored as 1 ml aliquots in a − 20 °C freezer that was only used for storage of samples. The freezer stands in a different room separated from the lab, PCR room and post PCR room. The urine samples from SLE patients and from pregnant women were kept in separate drawers in the − 20 °C freezer. The study was approved by the Regional Committees for Medical and Health Research Ethics (REK; reference number 2012/420). Fifty urine samples were obtained from a cohort of 30 relapsing remitting multiple sclerosis (RRMS) subjects, followed up at the Department of Human Neurosciences of Sapienza University of Rome and recruited between March 2016 and March 2018. All participants fulfilled the Italian Agency of Drug (AIFA) criteria for natalizumab treatment and the therapeutic protocol consisted of administration of 300 mg intravenous natalizumab every 4 weeks. The enrolled patients (12 males/18 females, mean age ± stand. dev: 30.2 ± 6.6; mean months of illness ± stand. dev. 85 ± 85.5; mean Expanded Disability Status Scale (EDSS) ± stand. dev. 1.9 ± 1.3) were visited before natalizumab treatment (baseline: 0 infusions) and after infusions at weeks 4, 8, 12, 16 and 20. Patients signed informed consent based on the approved Ethic Committee of Policlinico Umberto I of Rome (protocol number 130/13). Sixty-six urine specimens were collected from a cohort of 66 HIV-1-positive patients, admitted to the Infectious Diseases Clinic of the Polyclinic Tor Vergata Foundation from January 2019 to December 2019. Among the enrolled patients (55 males/11 females, age ranged from 21 to 76 years old: mean age ± stand. dev: 40.5 years old; median: 39.9 years old) 22 were new diagnoses naive to treatment and 44 were experienced patients on treatment with a triple-based antiretroviral regimen including protease/reverse transcriptase/integrase inhibitors. The study was approved by the local Ethic Committee of the University Hospital Tor Vergata (Rome, Italy) (protocol number 0027234/2018, 19 December 2018), and patients informed consent was ascertained. Healthy donors (n = 20), aged 18 ± 63 years, were recruited from blood donors attending the Italian Red Cross Blood Transfusion Centre in Rome (RMTC) and a sample of urine was obtained. Thirty urine specimens were collected from a cohort of 30 psoriasis patients, of both genders with a mean age of 33 ± 4 years, referred to the Dermatology Institute IDI IRCSS of Rome. One group of subjects, consisting of 15 patients, diagnosed with psoriasis vulgaris and the another 15 patients were diagnosed with psoriatic arthritis. These subjects did not show other autoimmune and chronic diseases. Informed consent was obtained from all patients.

### DNA purification and PCR

DNA was purified from 200 µl urine samples. The samples were centrifuged for 1 min at 12,000 g to remove cell debris. DNA purification was performed on cell-cleared urine using the QIAamp MinElute Virus Spin Kit according to the manufacturer’s instructions (Qiagen, Hilden, Germany; cat. No. 57704). DNA was eluted in 50 µl and 5 µl was used in PCR. The following primers were used to amplify a 299 bp fragment of LT: HPyV6LT.F: CAATGCATCACTACCTGGAC (nucleotides 4316-4335 in isolate 601a; HM011558) and HPyV6LT.R: GTTTGGGATTTCCGTTTGTG (nucleotides 4595-4614 in isolate 601a; HM011558). For HPyV7, a 344 bp fragment of LT was amplified using the primers HPyV7LT.F: CACGCAGGGCTTCCATATGG (nucleotides 4267-4282 in isolate 713a; HM011586) and HPyV7LT.R: GGTTTAAGAGCCTGCTGTTG-3′ (nucleotides 4591-4610 in isolate 713a; HM011586). The PCR conditions were 40 cycles of 30 s at 90 °C, 30 s at 55 °C, and 1 min at 72 °C. PCR was performed with Accustart II PCR SuperMix (Qunatabio, Beverly, MA, USA; cat. no. 95137-500). The plasmids pHPyV6-607a (Addgene, Watertown, MA, USA; cat. no. 24727) and pHPyV7-713a (Addgene; cat. no. 24728) were used as positive controls and to test the sensitivity of our PCR.

### Statistical analysis

HPyV6 and HPyV7 detection were summarized by counts and proportions. If continuous variables were normally distributed, they were expressed as mean ± SD; if not, they were expressed by median and range. The χ^2^ test was performed to evaluate differences in the viral detection and the Mann–Whitney U-test for non-normally distributed continuous variables was applied to analyse differences between patients. A *p* value < 0.05 was considered statistically significant.

## Results

To investigate whether other immunocompromised conditions may lead to viruria of HPyV6 and HPyV7, urine specimens of five cohorts were examined for the presence of DNA of these viruses. The cohorts included: systemic lupus erythematosus (SLE) patients, multiple sclerosis patients, HIV-positive patients, psoriasis patients and pregnant women. HPyV6 DNA was detected in 28.8% of the urine specimens from SLE patients and in 40.4% of the samples from pregnant women (Table 1). No HPyV6 could be amplified in urine from multiple sclerosis and psoriasis patients and from healthy blood donors. HPyV7 DNA was only detected in urine from SLE and pregnant women, although with a lower prevalence than HPyV6 (8.2% and 8.5%, respectively). The HPyV7-positive urine samples from SLE patient 3 were also HPyV6 positive. Two unique urine samples from SLE patient 4 and patient 5 contained both HPyV6 and HPyV7. Of the four HPyV7 positive urine samples from pregnant women, two were also positive for HPyV6. Six of the HIV-positive patients had HPyV6 viruria, but none had HPyV7 viruria. Interestingly, the six HIV patients with a HPyV6 positive urine sample were naïve, i.e., they had just started HAART treatment. HPyV6 and HPyV7 was not detected in any of the urine specimens from healthy blood donors, multiple sclerosis patients and psoriasis patients. No significant association was found between the presence of HPyV6 and HPyV 7 DNA versus age and gender when applicable, and HPyV6 and HPyV7 DNA versus disease (*p* < 0.05).

**Table 1:** DNA prevalence of HPyV6 and HPyV7 in different patient groups

Patient group (n)	HPyV6 (%)	HPyV7 (%)
SLE (5)		
SLE1 (5 samples)	1 (20.0)	0 (0)
SLE2 (7 samples)	1 (14.3)	0 (0)
SLE3 (13 samples)	4 (30.8)	2 (15.4)^a^
SLE4 (16 samples)	4 (25.0)	3 (18.8)^b^
SLE5 (32 samples)	11 (34.4)	1 (3.1)^c^
Total samples (73)	21 (28.8)	6 (8.2)
Multiple sclerosis (30)	0 (0)	0 (0)
Psoriasis vulgaris (15)	0 (0)	0 (0)
Arthritic psoriasis (15)	0 (0)	0 (0)
HIV-positive (66)	6 (9.1)	0 (0)
Pregnant women (47)	19 (40.4)	4 (8.5)^d^
Blood donors (20)	0 (0)	0 (0)

^a^These samples were also HPyV6 positive

^b^One sample was also HPyV6 positive

^c^This sample was also HPyV6 positive

^d^Two samples were also positive for HPyV6

## Discussion

HPyV6 and HPyV7 DNA has been detected in different body samples of immunocompromised patients [39, 43, 49–53], but viruria of these viruses has not been examined in SLE and MS patients and in pregnant women. We found that DNA of both viruses was detected in the urine from some SLE patient and pregnant women, with a higher prevalence of HPyV6 compared to HPyV7. A previous study failed to detect HPyV6 and HPyV7 DNA in skin from two SLE patients [39]. Similar to HPyV6 and HPyV7, intermittent episodes of urine shedding of BKPyV and JCPyV and simultaneous viruria of BKPyV and JCPyV was observed in SLE patients [70–75].

We could not detect HPyV6 or HPyV7 urine shedding in MS patients treated with natalizumab. CSF from 10 MS patients were negative for HPyV6 and HPyV7 DNA [42], suggesting that reactivation of these two HPyVs may be a rare event in MS patients. Studies by several groups have shown that BKPyV, JCPyV or both can be detected in urine from MS patients receiving natalizumab or β-interferon [76–81], indicating that HPyV viruria is not uncommon in these patients.

We did not discover HPyV6 and HPyV7 DNA in the urine samples of healthy blood donors. This is in agreement with a previous study that also failed to detect HPyV6 in the urine from 50 healthy volunteers [82] or in 189 urine specimens from symptomatic children and adults undergoing routine diagnostic testing [83]. A longitudinal study on 169 urine samples obtained from 32 organ transplant patients showed only one urine sample positive for HPyV7 DNA. This sample was obtained from a liver transplant child 8 months after transplantation [41].

The presence of HPyV6, but not HPyV7 DNA has been described in CSF, serum, and anal/rectal swabs from HIV-positive individuals [42, 43, 56], but neither HPyV6 nor HPyV7 were found in urethral samples or urine [56, 84]. We found that 6 of our 66 HIV-positive subjects displayed HPyV6 viruria, whereas Torres et al*.*, examined only 19 urine samples [84]. The relative small number of subjects in the latter study may explain the different findings with our results.

Hashida and colleagues examined nonlesional and lesional skin swabs from 30 psoriasis patients and reported that 58% (nonlesional) and 54% (lesional) samples were HPyV6 positive, while the HPyV7 prevalence was 42% and 25%. Of the skin swabs from healthy individuals, 14% were positive for HPyV6 and 6% were positive for HPyV7. The viral loads were also higher in both nonlesional and lesional samples of the psoriasis patients compared to the healthy individuals, indicating that HPyV6 and HPyV7 infection is higher in this inflammatory skin condition [85].

While PCR-based studies have shown that BKPyV and JCPyV viruria is common in pregnant women [86–89], less is known about urinal excretion of the novel HPyV. Cosma et al. [90] examine KIPyV, WUPyV and HPyV9 viruria in 100 non-pregnant and 100 pregnant women and found HPyV9 viruria in 2 non-pregnant and 3 pregnant women, whereas no KIPyV and WUPyV was detected in either cohorts. To the best of our knowledge, HPyV6 and HPyV7 viruria has not been investigated in pregnant women. We found that 40.4% of the women were positive for HPyV6 and 8.5% were positive for HPyV7. Two of the women (4.3%) had a urine sample that contained DNA of both viruses.

## Conclusions

In conclusion, immunodeficient conditions in SLE and HIV-positive patients may lead to viruria of HPyV6 and HPyV7, but whether these observations are linked to clinical conditions remain to be further explored. Our longitudinal study on SLE patients show that some of these patients may have intermitted episodes of HPyV6/7 reactivation.

## Data Availability

All data generated or analyzed during this study are included in this published article.

## Abbreviations

CSF: Cerebrospinal fluid
HPyV: Human polyomavirus
MS: Multiple sclerosis
VP: Viral protein
SLE: Systemic lupus erythematosus
